# Hospital efficiency in the eastern mediterranean region: A systematic review and meta-analysis

**DOI:** 10.3389/fpubh.2023.1085459

**Published:** 2023-02-02

**Authors:** Hamid Ravaghi, Mahnaz Afshari, Parvaneh Isfahani, Alireza Mahboub-Ahari, Victoria D. Bélorgeot

**Affiliations:** ^1^WHO Regional Office for the Eastern Mediterranean, Cairo, Egypt; ^2^School of Nursing and Midwifery, Saveh University of Medical Sciences, Saveh, Iran; ^3^Student Research Committee, Saveh University of Medical Sciences, Saveh, Iran; ^4^School of Public Health, Zabol University of Medical Sciences, Zabol, Iran; ^5^Department of Health Economics, Iranian Evidence Based Medicine Research Center, Tabriz University of Medical Sciences, Tabriz, Iran

**Keywords:** efficiency, meta-analysis, hospital, eastern mediterranean countries, technical efficiency (TE)

## Abstract

**Background:**

Recent rising costs and shortages of healthcare resources make it necessary to address the issue of hospital efficiency. Increasing the efficiency of hospitals can result in the better and more sustainable achievement of their organizational goals.

**Objective:**

The purpose of this research is to examine hospital efficiency in the Eastern Mediterranean Region (EMR) using data envelopment analysis (DEA).

**Methods:**

This study is a systematic review and meta-analysis of all articles published on hospital efficiency in Eastern Mediterranean countries between January 1999 and September 2020, identified by searching PubMed through MEDLINE, Web of Science, Scopus, Science Direct, and Google Scholar. The reference lists of these articles were checked for additional relevant studies. Finally, 37 articles were selected, and data were analyzed through Comprehensive Meta-Analysis Software (v.2.2.064).

**Results:**

Using the random-effects model, the mean hospital efficiency in Eastern Mediterranean hospitals was 0.882 ± 0.01 at 95% CI. Technical efficiency (TE) was higher in some countries such as Iraq (0.976 ± 0.035), Oman (0.926 ± 0.032), and Iran (0.921 ±0.012). A significant statistical correlation was observed between the hospital efficiency and the year of publication and sample size (*p* < 0.05).

**Conclusion:**

Efficiency plays a significant role in hospital growth and development. Therefore, it is important for healthcare managers and policymakers in the EMR to identify the causes of inefficiency, improve TE, and develop cost-effective strategies.

## Background

Countries across the Eastern Mediterranean Region (EMR) spent more than US$ 92 billion on their health in 2008. Being exposed to enormous substantial challenges such as increasing complexity and specialization, rapidly growing demand for new medical technologies, and social claims for high-quality services, the health system in EMR allocates 60–80% of its total budget to public hospitals (1, 2). Policymakers need to be ascertained that such an overwhelming investment is in line with society's real needs and preferences. Public hospitals are often viewed in terms of the efficient use of public resources, where the final objective of these prominent non-market sectors goes beyond that of the free market such as income or benefit margin. According to the World Health Organization (WHO), hospital performance in the EMR is often poor due to several reasons such as mismanagement, low bed occupancy rate, long average lengths of stay, and high rate of hospital-associated infections (3). In its 2009 report, the WHO highlighted that hospital resources are inefficiently utilized in low- and middle-income countries compared to their developed counterparts (3). Given the complex nature of functions undertaken by public hospitals and the absence of usual market indicators, there is a clear necessity for appropriate performance measurement tools to seek out best practices and identify gaps for improvement (4, 5).

A wide variety of analytic methods has been utilized by researchers to measure hospital efficiency in terms of costs and production frontiers and the associated inefficiency of individual organizations (6–8). These techniques can be divided into two main categories: parametric and non-parametric methods. Parametric methods use econometric techniques to estimate the parameters of a specific cost of production functions, and non-parametric methods use observed real-world data to draw the shape of the frontier (5). The premier of parametric methods in use is called stochastic frontier analysis (SFA) which uses multivariate regression analysis to estimate a cost or production function, where the decomposed unexplained error term represents inefficiency (which, in the case of a cost function, will always be greater than zero) (5).

Most non-parametric methods take the form of data envelopment analysis (DEA) and its many variants. DEA uses linear programming methods to infer a piecewise linear production possibility frontier in effect seeking out those efficient observations that dominate (or envelop) the others. In contrast to parametric methods, DEA can handle multiple inputs and outputs without difficulty. DEA determines a best practice frontier of various decision-making units (DMUs) that envelops all inefficient DMUs. The estimation of the technical efficiency score is the major concern of almost all DEA models, indicating that the proper allocation of resources is not part of the calculations. Compared to parametric methods that need to initially specify production function before measurement, DEA is not subject to production function specification (9, 10).

In recent years, a vast amount of studies has been conducted in high-income countries benefiting from cutting-edge methodologies (8, 11), so some of them incorporated preferences into the analysis (7, 12, 13), as well as in the EMR, aiming at measuring hospital efficiency through both parametric and non-parametric approaches (14–16). A context-specific overview and analysis of existing articles are helpful for everyone interested in the field of efficiency measurement in healthcare with a focus on hospitals. According to our preliminary search, two systematic reviews have been conducted to address the issue in the hospital setting (17, 18). The study by Ravaghi et al. explored the potential sources of inefficiency in EMR hospitals which had been reported by 56 eligible studies and summarized the possible solutions by using qualitative synthesis (18). The second review has included 22 eligible studies from the Gulf region and estimated the technical efficiency (TE) through pooled estimation. Despite this study having systematically reviewed the existing literature and addressed one important aspect of hospital economic performance, the focus of the study was only on Gulf region countries which might limit the generalizability of the study findings to other similar settings (17). This systematic review aimed to deeply scrutinize the published literature on hospital efficiency in EMR hospitals and estimate technical efficiency which has been reported by previous studies through meta-analysis.

## Methods

The present study is a systematic review and meta-analysis to examine hospital efficiency in the EMR using DEA.

### Eligibility criteria

Studies were included in this systematic review if they (1) measured efficiency using a statistical method, (2) used the hospital as the analysis unit, (3) measured hospital efficiency using data envelopment analysis, (4) reported data necessary to calculate it, (5) were written in English, (6) performed a study in EMR, (7) contained data required for analysis (by access to the full text or by request from the author), and (8) included mean and SD (VRS TE or CRS TE).

Studies were excluded if they (1) used methods other than DEA (for example SFA and Pabon Lasso Model), (2) are performed at private hospitals or settings other than a hospital, and (3) were a thesis, case series, randomized controlled trials, case-control, commentaries, letters to the editor, book chapters, books, editorials, expert opinions, brief reports, and reviews.

### Search sources and search strategies

PubMed through MEDLINE, Web of Science, Scopus, Science Direct, and Google Scholar were searched from January 1999 to September 2020. All of the keywords were in English, and the search strategy was restricted to English-language publications. The electronic search was complemented by hand-searching of the related articles as well as the reference lists of the final studies (Table 1).

**Table 1:** Search strategy specific to the international electronic databases.

**Databases**	**Search strategy**	**Preliminary searches**
PubMed	((((“Efficiency”[mesh] OR “Productivity”[mesh] OR “Organizational Efficiency”[tiab] OR “Data Envelopment Analysis” OR “inefficiency”[tiab] OR “Productivity, Organizational”[tiab] OR “Organizational Productivity”[tiab] OR “Program Efficiency”[tiab] OR “Efficiency, Program”[tiab] OR “Efficiency, Administrative”[tiab] OR “Administrative Efficiency”[tiab] OR “Efficiency”[tiab] OR “Data Envelopment Analysis”[tiab] OR “Pabon Lasso”[tiab] OR “Stochastic Frontier Analysis”[tiab] OR “Productivity”[tiab])) AND (“Hospital”[mesh] OR “hospital”)) AND (“Afghanistan” OR “Bahrain” OR “Djibouti” OR “Egypt” OR “Iran (Islamic Republic of)” OR “Iraq” OR “Jordan” OR “Kuwait” OR “Lebanon” OR “Libya” OR “Morocco” OR “Oman” OR “Pakistan” OR “Qatar” OR “Saudi Arabia” OR “Somalia” OR “Sudan” OR “Syrian Arab Republic” OR “Tunisia” OR “United Arab Emirates” OR “Yemen” OR “Palestine”))	1009
Scopus	**(** *TITLE-ABS-KEY* **(** “*Data Envelopment Analysis”* **OR** *efficiency* **) AND** *TITLE-ABS-KEY* **(** “*Afghanistan”* **OR** “*Bahrain”* **OR** “*Djibouti”* **OR** “*Egypt”* **OR** “*Iran (Islamic Republic of)”* **OR** “*Iraq”* **OR** “*Jordan”* **OR** “*Kuwait”* **OR** “*Lebanon”* **OR** “*Libya”* **OR** “*Morocco”* **OR** “*Oman”* **OR** “*Pakistan”* **OR** “*Qatar”* **OR** “*Saudi Arabia”* **OR** “*Somalia”* **OR** “*Sudan”* **OR** “*Syrian Arab Republic”* **OR** “*Tunisia”* **OR** “*United Arab Emirates”* **OR** “*Yemen”* **OR** “*Palestine”* **) AND** *TITLE-ABS-KEY* **(** *hospital* **)** **) AND (** *LIMIT-TO* **(** *LANGUAGE*, “*English”* **)** **)**	391
Web of Science	(( TS= (“Data Envelopment Analysis” OR efficiency ) AND TS= (“Afghanistan” OR “Bahrain” OR “Djibouti” OR “Egypt” OR “Iran (Islamic Republic of) ” OR “Iraq” OR “Jordan” OR “Kuwait” OR “Lebanon” OR “Libya” OR “Morocco” OR “Oman” OR “Pakistan” OR “Qatar” OR “Saudi Arabia” OR “Somalia” OR “Sudan” OR “Syrian Arab Republic” OR “Tunisia” OR “United Arab Emirates” OR “Yemen” OR “Palestine”) AND TS= ( hospital ) )) AND LANGUAGE: (English) AND DOCUMENT TYPES: (Article) Indexes=SCI-EXPANDED, SSCI, AandHCI, CPCI-S, CPCI-SSH, ESCI Timespan=All years	172
Google Scholar	“*Data Envelopment Analysis” **OR** efficiency AND* “Afghanistan” OR “Bahrain” OR “Djibouti” OR “Egypt” OR “Iran (Islamic Republic of)” OR “Iraq” OR “Jordan” OR “Kuwait” OR “Lebanon” OR “Libya” OR “Morocco” OR “Oman” OR “Pakistan” OR “Qatar” OR “Saudi Arabia” OR “Somalia” OR “Sudan” OR “Syrian Arab Republic” OR “Tunisia” OR “United Arab Emirates” OR “Yemen” OR “Palestine”))	2200
Science Direct	(“Data Envelopment Analysis“ OR efficiency) AND “Eastern Mediterranean countries” AND (hospital)	24

### Screening and study selection

Search results were imported and managed *via* EndNote X8 (Thomson Reuters, New York, USA). Duplicates were first removed electronically and then manually. Subsequently, the title and abstract of the included studies were independently screened by two reviewers (AM and MA), and disagreements were finally resolved by helping a third reviewer (HR). The full text of potential studies was retrieved and reviewed by the two reviewers. Email or ResearchGate contact was used to obtain full-text or English versions of the inaccessible studies.

### Data extraction

Two reviewers (MA and AM) extracted data for the country where the study was conducted, year of publication, research purpose, sample size, data collection method, number of hospitals examined, and mean and standard deviation (SD) of TE.

### Quality assessment

The methodological quality of the eligible studies was assessed using the five-question instrument which was introduced and applied by Mitton et al. (19) (see the Appendix). Each question was given a score of 0 (not present or reported), 1 (present but low quality), 2 (present and mid-range quality), or 3 (present and high quality). Criteria for assessment of quality included a literature review and identifying research gaps; research questions, hypotheses, and design; population and sampling; data collection process and instruments; and analysis and reporting of results. The assessment was conducted by both AM and MA, and discrepancies were then resolved either by discussion or by the third reviewer (HR).

### Data analysis

Since the mean and standard deviation of TE had not been reported by most of the included studies, we dealt with this missing information by contacting the authors of these studies or calculating the values using available data. Meta-analysis was conducted to synthesize the mean technical efficiency (TE) using the random-effects model by the sample size weighting (20). The results were presented with 95% confidence intervals (95% CIs) (20). Statistical heterogeneity among the studies was assessed by Cochran's Q statistic and I2 index (21, 22). As the analytical results revealed a high heterogeneity (96.07%), the random-effects model was employed and covariates between variables were examined using the meta-regression function. All these statistical analyses were conducted using the Comprehensive Meta-Analysis Software (v.2.2.064).

## Results

**Figure 1:**
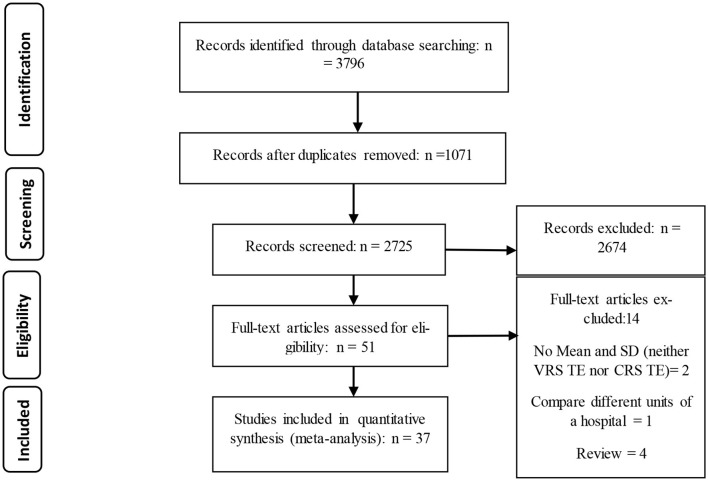
PRISMA flow diagram (33) illustrating the study selection process.

The initial search resulted in 3,796 articles. After excluding duplicates and irrelevant articles, 2,725 studies were selected for abstract examination, whereas 2,674 articles were removed after reviewing abstracts. We also scrutinized 51 full-text articles for eligibility and excluded 14 because they did not satisfy our inclusion/exclusion criteria [Four were review articles (17, 18, 23, 24), five used different estimation methods (14, 16, 25–29), one article was conducted in a single hospital ward (30), and two articles did not report mean and SD (neither VRS TE nor CRS TE) (31, 32)]. Finally, 37 articles were found eligible for inclusion in this systematic review and meta-analysis. The reference lists of these 37 articles were manually searched, but no additional studies were included (Figure 1). The PRISMA flow diagram (33) was followed in this study.

### Characteristics of the included studies

**Figure 2:**
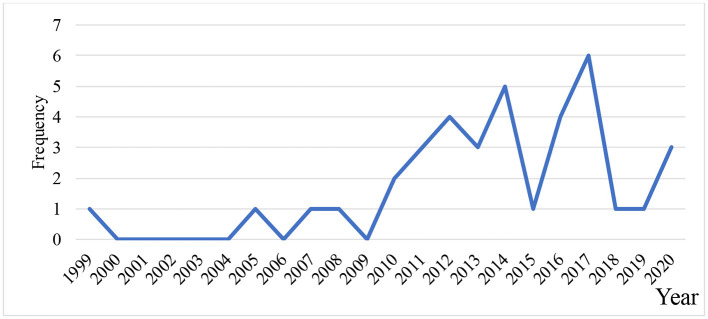
Distribution of hospital efficiency studies by publication year.

Over half of the studies had been published after 2010, with most having been conducted in 2017 and 2014 (Figure 2). Studies were only conducted in 11 of the 22 EMR countries. The overwhelming majority of these are located in Iran (*N* = 20) and Saudi Arabia (*N* = 4). The sample size varied from three (34) to 270 (35) hospitals. Health reports, interviews, hospital records, or annual statistical records were reported as the sources of data.

Efficiency had been assessed in light of various concepts including technical, scale, and pure efficiency with a primary focus on TE in the reviewed studies. The reviewed studies varied in the models used to estimate the TE of public hospitals. Twelve studies used both constant and variable return to the efficiency scale (CRS and VRS), whereas 19 applied variable return to scale (VRS) and 6 used constant return to scale (CRS). The inputs used in the included studies are presented in Table 2, with a range of 2–5. Predominant inputs were the labor (including full-time and part-time physicians, full-time and part-time nurses, midwives, non-medical staff, and dentists) and capital (number of beds) variables. Two studies (36, 37) used capital expenses in the inputs. Numerous output dimensions were used in the efficiency models (range: 1–9 variables). Output variables focused on the number of outpatient visits and inpatient admissions. Twelve studies used bed turnover (BTR) and occupancy (BOR) rates, and 10 studies used an average length of stay (ALS), while one study (38) used mortality rate in hospitals as an output variable (Table 2).

**Table 2:** Characteristics of included studies reported technical efficiency in hospitals located in EMR.

**Author**	**Year**	**Country**	**VRS TE**		**CRS TE**		**Sample Size**	**DEA orientation model;**	**Returns-to-scale**	**Source of data**	**Inputs**	**Outputs**	**Quality**
			**Mean**	**SD**	**Mean**	**SD**							**score**
Al-Shammari (39)	1999	Bahrain	NR^*^	NR	0.584	0.266	15	Optimization modeling system for linear programming LINDO^*^;	CRS^*^	The Annual Statistical Reports	Number of bed days, physicians, health workforce	The number of inpatient days, minor operations, major operations	
Ramanathan (15)	2005	Oman	0.926	0.145	0.873	0.173	20	Frontier analysis (Malmquist Index);	Using two methods: CRS and VRS^*^	from the Annual Statistical Abstract published by the Ministry of National Economy, the Annual Health Report published by the MoH^*^	The number of beds, physicians, and other medical workforces.	Number of visits, in-patient services, surgical operations	14
Hajialiafzal et al. (40)	2007	Iran	0.904	0.146	NR	NR	53	Input-oriented model;	VRS	the Annual Statistical Report published by the SSO^*^	Total number of FTE^*^ medical doctors, FTE nurses, of other FTE workforces, number of beds	Number of outpatient visits and emergency visits, ratio of major surgeries to total surgeries, total number of medical interventions and surgical procedures	14
Hatam (36)	2008	Iran	0.966	0.083	NR	NR	18	Non-parametrical estimation	-	The data collected, in field and library studies, interview and referring to available documents	Number of beds, FTE, total expenses	Patient-days, BOR^*^, BTR^*^, ALS^*^, ratio of available beds to constructed beds, hoteling expenses, bed-day costs, workforce costs	12
Jandaghi et al. (41)	2010	Iran	0.968	0.64	0.95	0.051	8	-	Using two methods: CRS and VRS	medical documents of hospitals	Number of physicians, nurses, medical workforce, official workforce, annual costs of hospital	Numbers of clinical visits, emergency visits, and bed days	14
Hatam et al. (42)	2010	Iran	0.93	0.013	NR	NR	21	Malmequist index;	VRS	The data were collected using interviews, questionnaires, and available documents	Number of hospital beds, FTE physicians, nurses, and other workforces	BOR, patient–day admissions, bed days, ALS, BTR	13
Shahhoseini et al. (43)	2011	Iran	0.97	0.64	0.91	0.15	12	Input-orientated method;	Using two methods: CRS and VRS	A questionnaire was used to gather information	number of active beds, nurses, physicians, non-clinical workforce	Number of surgeries, outpatients visits, BOR, ALS, inpatient days	15
Ketabi (44)	2011	Iran	0.967	0.093	NR	NR	23	Input-oriented model;	VRS	reports submitted to the Care Deputy of the Medical University of Isfahan	Beds, human resources	inpatient days, outpatient days, number of surgeries, BOR	14
Kiadaliri (45)	2011	Iran	0.913	0.102	NR	NR	19	Input-oriented model;	VRS	checklists	Beds, human resources	Inpatient days, outpatient days, number of surgeries, BOR	15
Osmani (46)	2012	Afghanistan	0.883	0.136	0.691	0.242	68	Input-oriented model;	Using two methods: CRS and VRS	Afghanistan's Health Management Information System (HMIS) database has provided the required data.	Numbers of physicians, midwives, nurses, non-medical workforce, and beds	Number of outpatient visits, inpatient admissions, and patient days, ALS, BOR, number of hospital beds, bed-physician and outpatient physician ratio, number of physicians	14
Chaabouni and Abednnadher (47)	2012	Tunisia	NR	NR	0.974	0.048	10	Output-oriented model;	CRS	from the health ministry and reports of the national institute of statistics	numbers of physician, nurses, dentists and pharmacists, other workforces, beds	Number of outpatient visits, admissions, post-admission days	14
Farzianpour et al. (48)	2012	Iran	0.946	0.067	0.988	0.04	16	Output-oriented model;	using two methods: CRS and VRS	gathered through interviewing department of health and human resources management and support assistant	Number of physicians, practicing nurses in health facilities, and active beds	Numbers of inpatients, outpatients, ALS	14
Sheikhzadeh et al. (49)	2012	Iran	0.79	0.24	NR	NR	6	-	VRS	checklist, interview, documental profiles review: the regulation booklet of MoH, booklets, professional magazines, annual reports of creditable domestic and international organizations such as the WHO and UNDP, internet sources	Numbers of specialist physicians, general physicians, nurses, residents, medical team workforce with a degree (Bachelor's), medical team, nonmedical and support workforce, and active beds	Numbers of emergency patients, outpatients, and inpatients, average daily inpatients residing in hospital	15
Yusesfzadeh et al. (50)	2013	Iran	0.584	0.266	NR	NR	23	Input-oriented model;	VRS	Available documents in hospitals	Number of active beds, doctors, and other workforces	Number of outpatient admissions and bed days	13
Ajlouni et al. (51)	2013	Jordan	_	_	0.939	0.272	15	-	CRS	Annual Statistical Reports	numbers of bed days, physicians per year, and health workforce per year	Patient days, numbers of minor operations and major operations	14
Abou El-Seoud (52)	2013	Saudi Arabia	0.846	0.139	NR	NR	20	Input and output-oriented model;	VRS	data from the MoH's Statistical Yearbook	Numbers of specialists, nurses, allied workforce, beds	Numbers of visits, patient hospital admissions, laboratory tests, beneficiaries of radiological imaging	14
Rasool et al. (53)	2014	Pakistan	0.786	0.441	0.703	0.249	16	Input-orientated method;	Using two methods: CRS and VRS	Not Reported	Number of beds, specialists, nurses	Number of outpatient visits, inpatient admissions, and total number of surgeries	14
Torabipour et al. (54)	2014	Iran	0.996	0.162	NR	NR	12	Input-oriented model;	VRS	Medical records and documents of the hospitals	Number of nurses, number of occupied beds and number of physicians.	Number of outpatients and inpatients, the average of hospital stays, and number of major operations	12
Mehrtak et al. (55)	2014	Iran	0.809	0.242	NR	NR	18	Input-oriented model;	VRS	Hospitals' monthly performance forms	Numbers of active beds, physicians, nurses, discharged patients	Number of surgeries and discharged patients, BOR	11
Lotfi et al. (56)	2014	Iran	NR	NR	0.924	0.105	16	Input-oriented model;	CRS	Were collected through separate special checklists	Number of physicians, nurses, other workforces, active beds	BOR, numbe of patients and surgeries	12
Askari et al. (57)	2014	Iran	0.956	0.052	NR	NR	13	Input-oriented model;	VRS	Direct observation, interviews and referring to the existing documents and statistics of hospitals' activities	Numbers of active beds, nurses, physicians, non-clinical workforce	Hospitalization admissions, BOR (%), and number of surgeries	11
Shetabi et al. (58)	2015	Iran	0.876	0.199	NR	NR	7	Minimizing production factor model;	VRS	Library and field study	Number of active beds, doctors, nurses, and other workforces	Numbers of accepted inpatients, outpatients and BOR (%)	14
Mahate and Hamidi (59)	2016	united arab emirates	0.659	0.16	0.523	0.204	96	Output-oriented model;	Using two methods: CRS and VRS	Not Reported	Numbers of beds, doctors, dentists, nurses, pharmacists and allied health workforce, administrative workforce	Numbers of treated inpatients, outpatients, ALS	15
Kalhor et al. (60)	2016	Iran	0.819	0.188	NR	NR	54	Input-oriented model;	VRS	A checklist was developed by researchers based on extensive literature reading.	Total numbers of FTE medical doctors, nurses, supporting medical workforce including ancillary service workforce, and beds	Number of patient days, outpatient visits, patients receiving surgery, ALS	15
Kakeman et al. (61)	2016	Iran	0.821	0.188	NR	NR	54	Input-oriented model;	VRS	The checklist used to collect data	Number of active beds, physicians, nurses, and other medical workforces	Number of outpatient visits, surgeries, and hospitalized	15
Nabilou et al. (62)	2016	Iran	0.992	0.019	NR	NR	17	Input-oriented model;	VRS	Checklists developed by the researchers	Active beds, nurses, doctors and other workforces	Outpatient admissions,bed days, number of surgical operations	13
Farzianpour et al. (63)	2017	Iran	0.818	0.207	NR	NR	19	Input-oriented model;	VRS	A questionnaire that contained the profile of the hospital, required variables	Number of physicians, total workforce, and active beds	Number of outpatients and BOR	12
Sultan and Crispim (64)	2017	Jordan	NR	NR	0.839	0.175	27	Input-oriented model;	CRS	Statistical report	Numbers of beds, physicians, healthcare workforce, administrative workforce	Inpatient days, outpatient visits, emergency departments, and ambulances	14
AlyHelal and Elimam (65)	2017	Saudi Arabia	0.923	0.1	0.947	0.09	270		using two methods: CRS and VRS	Health Statistical Annual Book	Numbers of beds, doctors, nurses, and allied medical workforce	Numbers of individuals visiting admitted patients, radiography service beneficiaries, laboratory testing beneficiaries, and inpatients	14
Arfa et al. (37)	2017	Tunisia	0.92	0.11	NR	NR	105	Output-oriented model;	VRS	Data were collected from various MoH reports for 2010 and from a survey for 2000.	Number of physicians, surgical dentists, midwives, nurses and equivalents, and beds, operating budget	Outpatient visits in stomatology wards, outpatient visits in emergency wards, outpatient visits in external	13
Kassam (34)	2017	Iraq	NR	NR	0.976	0.061	3	Input and output-oriented model; LPI^*^	CRS;	Not Reported	numbers of doctors, nurses, and other health workforces	Numbers of outpatients, laboratory tests, radiology tests, sonar tests, emergency visits	13
Mousa and Aldehayyat (35)	2018	Saudi Arabia	0.964	0.119	0.918	1.8	270	Input-oriented model;	using two methods: CRS and VRS	Health Statistical Annual Book	Number of physicians, nurses, pharmacists, allied health professionals, beds	Number of outpatient visits, inpatients, laboratory investigations, X-rays patients, X-rays films, total number of surgical operations	14
Migdadi and Al-Momani (66)	2018	Jordan	0.993	0.019	NR	NR	15	Input-oriented model;	VRS	The annual statistical reports of the MoH	Number of physicians, nurses, beds	ALS, number of surgeries, BOR	15
Kakeman and Dargahi (67)	2019	Iran	0.897	0.103	0.839	0.12	42	Input-oriented model;	using two methods: CRS and VRS	The statistical centers of Universities of Medical Sciences	The number of doctors, nurses, and other staff, and the number of hospital beds	The number of outpatients, emergency department visits, the number of inpatient days	13
Alatawi et al. (38)	2020	Saudi Arabia	0.87	0.18	0.76	0.23	91	Input-oriented model;	Using two methods: CRS and VRS	Official statistical, informational and research databases of administration of statistics, information and administration of research and studies	Number of hospital beds, number of physicians, nurses and allied health personnel.	The number of outpatient visits, discharged patients, surgical operations, radiological and laboratory tests and hospital mortality rate	13
Alsabah et al. (68)	2020	Kuwait	1	0.18	0.829	0.2	15	Input-oriented model;	Using two methods: CRS and VRS	“Health, Kuwait” annual report published by the MOH'	The number of beds, total number of doctors, nurses, non-medical workers.	Total outpatient visits, total number of discharges	14
Alwaked et al. (69)	2020	Jordan	0.697	0.225	NR	NR	29	Output-orientated model;	VRS	MoH Annual Statistical Book	Beds, Physician, Medical Staff, Non-medical staff	Inpatient	13

^*^CRS, constant return to scale; VRS, variable return to scale; MoH, Ministry of Health; LINDO, Linear, Interactive, Discrete Optimizer; BOR, bed occupancy rate; BTR, bed turnover rate; ALS, average length of stay; FTE, full-time employee; LPI, Luenberger productivity indicator; SSO, The Social Security Organization; NR, not reported.

### The methodological quality of included studies

No articles were excluded based on the quality appraisal. All the included studies acquired more than 70% of the overall score. So that 95% (*N* = 35) of the studies were in the third quarter Q3 (≥75% of overall score). More than 65% (*N* = 13) of the studies have developed a good research question, and most of them adopted an appropriate sample size (92%, *n* = 34). With respect to the data collection method, 100% of the studies followed the standard guideline in collecting data and acquired the full score in this item. The analysis and results of the reporting item were the one item that most of the studies could not get a full score; therefore, only 33% (*N* = 12) of the studies got a full score here. The quality assessment scores are presented in Table 2.

**Figure 3:**
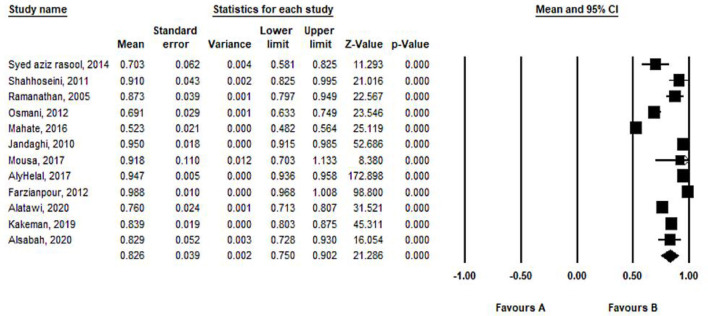
Mean and standard deviation of TE with CRS model in included studies based on the random-effects model.

To examine the consistency of efficiency assessments, we conducted a meta-analysis of the estimated TE scores reported in the reviewed studies. The mean and standard deviation of TE with the CRS model in Eastern Mediterranean hospitals are 0.826 ± 0.03 at the 95% significance level. According to the random-effects model, TE was higher in Iran (0.988 ± 0.010) in 2012 (Figure 3).

**Figure 4:**
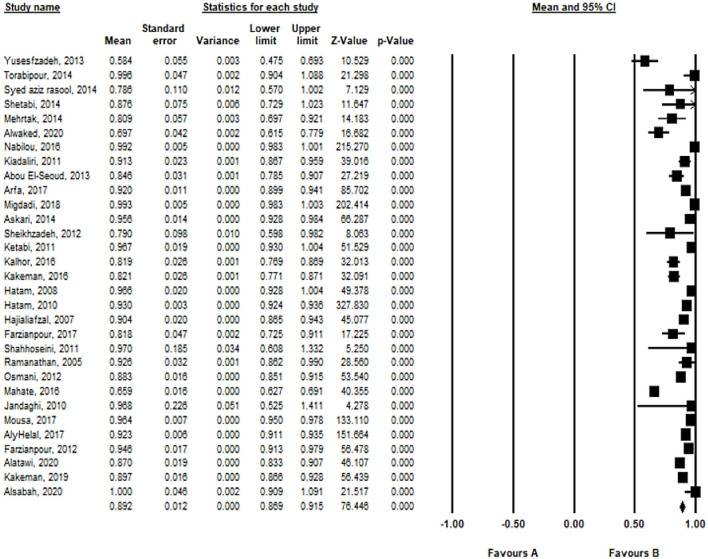
Mean and standard deviation of TE with the VRS model in included studies based on the random-effects model.

The mean and standard deviation of TE with the VRS model in Eastern Mediterranean hospitals are 0.892 ± 0.012 at a 95% significance level. According to the random-effects model, TE was high in Kuwait (1.00 ± 0.046) (Figure 4).

Studies examining fewer hospitals for estimations reported higher efficiency scores compared to studies using more hospitals. Studies published in lower-middle-income countries reported TE to score higher compared to others (Table 3).

**Table 3:** Subgroup analysis of efficiency scores by country and method of analysis.

**Variable**			**Number of studies**	**Mean and standard error**	**95% CI**	**I^2^**	***P*-value**
VRS	Countries	High income	7	0.881 ± 0.036	0.810-0.953	98	≤0.0001
		Upper-middle income	2	0.848 ± 0.14	0.558–1.138	98	≤0.0001
		Lower-middle income	21	0.904 ± 0.013	0.879–0.928	92.4	≤0.0001
		Low income	1	0.883 ± 0.016	0.851–0.915	–	–
	Number of hospitals	30≤	10	0.867 ± 0.024	0.820-0.915	97.3	≤0.0001
		<30	21	0.913 ± 0.013	0.888–0.939	93.8	≤0.0001
CRS	Countries	High income	6	0.804 ± 0.090	0.629–0.980	98.8	≤0.0001
		Upper-middle income	–				
		Lower-middle income	5	0.891 ± 0.039	0.815–0.967	94	≤0.0001
		Low income	1	0.691 ± 0.029	0.633–0.749	–	–
	Number of hospitals	30≤	6	0.774 ± 0.078	0.622–0.927	99	≤0.0001
		<30	6	0.894 ± 0.031	0.832–0.955	86.8	≤0.0001

The results of the heterogeneity test indicated a high level of heterogeneity between the studies (I^2^ = 96.07%, *P* = 0.0001). Therefore, potential sources of heterogeneity were examined using the meta-regression function. The results are displayed in Table 4, indicating that the year of publication and sample size of articles have caused heterogeneity between the reviewed studies (*p* < 0.05). The results of meta-regression with VRS, based on the year of study, demonstrated that an increase of one unit per year of study causes a higher incidence of hospital efficiency by 0.003 units. Moreover, the efficiency of the hospital decreases by 0.00008 as the sample size of articles increases. On the other hand, the results of meta-regression with CRS, based on the year of study, demonstrated that an increase of one unit per year of study causes a lower incidence of hospital efficiency by 0.006 units. Moreover, the efficiency of the hospital decreases by 0.006 as the sample size of articles increases.

**Table 4:** Results of the heterogeneity test (meta-regression model).

**Variables**		**Coefficient**	**SE**	***P*-value**
VRS	Publication year	0.003	0.0005	≤0.0001
	Sample size	−0.00008	0.00002	≤0.0001
CRS	Publication year	−0.006	0.001	≤0.0001
	Sample size	−0.006	0.008	≤0.0001

## Discussion

Several systematic reviews have been conducted on hospital efficiency worldwide (18, 70, 71). For example, a 2018 study reviewed 57 articles using DEA (18), and a 2014 study reviewed 23 articles using DEA, SFA, and balanced scorecard (71). To our knowledge, this is the first attempt to measure hospital efficiency using meta-analysis in the Eastern Mediterranean region. There was a growing trend in recent years to measure the efficiency of hospitals using different methods. In this study, we reviewed studies that measured the TE of hospitals in EMR countries. A total of 37 articles which calculated hospital efficiency using DEA were eligible for inclusion in the meta-analysis.

It must be noted that the vast majority of studies on hospital efficiency were conducted in Iran. This may partly be due to the Iranian Ministry of Health and Medical Education's attempt at reducing hospital costs. In addition, efficiency and strategies for improving it have become a key priority for the Iranian government.

A mean TE of 0.882 ± 0.01 was estimated for Eastern Mediterranean countries. This finding is consistent with the results of previous studies in other countries (24, 72, 73). Pereira et al. (4) examined the convergence in productivity and indicated that in the EMR, the performance spread among countries is decreasing and the gap between the best and worst practice frontier is increasing. Also, they showed that innovator EMR countries are Egypt, Jordan, Kuwait, Qatar, Tunisia, and the United Arab Emirates, and the lagging EMR WHO Member State is Somalia. In the study conducted by Du (73) on Chinese hospitals economic performance, the mean hospital efficiency was estimated at 0.74, 0.902, and 0.805 in the Central, Eastern, and Western regions of the country, respectively (73). Blatnik et al. (72) examined hospital efficiency in Slovenia and reported a mean TE of 0.936 (72). These extensive empirical works indicate that hospital efficiency can significantly vary across different countries and regions (4, 11).

According to our findings, the mean hospital efficiency varied in high-income countries such as Saudi Arabia, Oman, the United Arab Emirates, and Bahrain. For example, Oman had the highest mean TE, and Bahrain had the lowest mean TE. According to the 2017 WHO's report on “Eastern Mediterranean Region Framework for health information systems and core indicators for monitoring the health situation and health system performance,” Bahrain and Oman had the highest general government expenditure on health as a percentage of general government expenditure (10.5 and 6.8%, respectively) among the four countries (74). On the other hand, mean hospital efficiency also varied in low- and middle-income countries such as Pakistan, Afghanistan, Iran, Jordan, Tunisia, Palestine, and Iraq. For instance, among these countries, Iraq and Iran stood at the top of the list, whereas Pakistan had the lowest mean TE among other counterparts. WHO's world health report 2017 highlighted that among these seven countries, Iran had the highest and Pakistan had the lowest general government expenditure on health as a percentage of general government expenditure (17.5 and 4.7%, respectively) (74). Therefore, hospital managers and policymakers must focus on improving efficiency and reducing healthcare costs in regions that have lower rates of hospital efficiency. Furthermore, a study using the ‘Sustainable Public Health Index' showed that Bahrain, Egypt, Iran (the Islamic Republic of), Jordan, Kuwait, Libya, Morocco, Oman, Pakistan, Qatar, Saudi Arabia, the Syrian Arab Republic, and the United Arab Emirates were the efficient EMR countries between WHO Member States (75).

Hospital internal structure (11), regional differences (4, 11), and decision-maker participation in the assessment (13) of the environmental, social, and economic sustainability of the hospital (7) have a significant impact on the efficiency of hospitals. The development of outpatient care (23), reducing supplier-induced demand (76), the strengthening of hospital management and quality management (70, 77), the strengthening of governance and regulation (78), and enhanced crisis resilience such as COVID-19 crisis (8) are recommended as effective strategies to increase hospital efficiency. In addition, hospitals can serve as productive business entities through health system structure reform at the macro level, proper implementation of healthcare stratification, and responsiveness of insurance companies (23, 79). This allows hospitals to increase patient satisfaction and provide safe, high-quality care.

The measurement of hospital efficiency is done through a set of input and output variables (80, 81). The present findings show that the most commonly used input variables in studies on hospital efficiency in the EMR are the number of employees and the number of beds, while the most commonly used output variables are the number of outpatient visits, the number of inpatient admission, and the number of operations. For example, in a study on hospital efficiency in Oman, Ramanathan (2005) used outpatient visits, inpatient services, and surgical operations as outputs, and the number of beds and manpower as inputs (15). In addition, some studies have used other inputs such as work hours, non-labor costs (i.e., equipment, food, and drugs), the area of the hospital in cubic meters (82, 83), and outputs such as mortality rate, number of nursing students, number of medical students, number of nursing and medical training weeks, and number of scientific publications (84, 85). Pereira proposed a framework to make a “sustainable public health index” and assessed the performance of the WHO Member States by using the 13 indicators of the UN's SDG 3 targets as input and output (75). They found that the EMR was in fourth place among six WHO regions (75). Researchers must use more input and output variables when measuring hospital efficiency to increase the accuracy of their findings.

In some countries, mean efficiency has increased significantly in recent years. For example, Helal et al. (65) showed a significant improvement in the average efficiency of Saudi hospitals in 2014 compared to 2006, with hospital efficiency reaching 92.3% in 2014 (65).

The present systematic review showed that, on average, small-scale (47) and public hospitals (61) have a lower level of efficiency. For example, Chaabouni and Abednnadher (2016), who examined Tunisian public hospitals, reported a positive association between cost-effectiveness and hospital size. They found that the mean cost-effectiveness was 0.995 in large hospitals compared to 0.875 in small hospitals (47). In a study on Iranian hospitals, Ketabi (2011) showed that CCUs in 83.3% of teaching hospitals and 60% of private hospitals perform inefficiently (44). This was attributed to the excess of medical equipment as well as personnel and technological capabilities. Teaching hospitals were less efficient because of bureaucratic processes, and private hospitals had lower BORs. There is a larger demand for care in public hospitals than in private hospitals, and thus, public hospital managers in particular must make optimal use of their resources.

The present review showed that hospital efficiency decreases by 0.00008 as the sample size of articles increases. On the other hand, hospital efficiency increases by 0.003% as the publication date increases by 1 year. In other words, the time sequence of studies on hospital efficiency indicates lower levels of efficiency in recent years compared to previous years.

## Conclusion

The results of this systematic review and meta-analysis of hospital efficiency in Eastern Mediterranean countries highlighted that the reviewed studies varied in the model used to estimate the technical efficiency in public hospitals (CRS and VRS). The EMR studies have based their analysis on hospital inputs. Also, a significant statistical correlation was observed between the hospital efficiency and the year of publication and sample size.

The results of this article should, however, be cautiously interpreted. Although the pooled estimation of hospital efficiency reflects only the performance of a limited number of Eastern Mediterranean countries, this gap in the literature indicates that the reviewed studies are not comprehensive in terms of coverage and methodology. Other variables, such as ownership or type of hospital, can impact the results of efficiency analysis, but a small sample size restricts control of this variable.

In recent years, the number of studies on efficiency has significantly increased, likely due to the increase in interest in the subject due to resource scarcity. To enable effective and efficient hospital management and improvement in hospital efficiency, health managers and policymakers must identify the causes of hospital inefficiency. An effective way of increasing hospital efficiency is by using evidence-based interventions. Therefore, health policymakers in Eastern Mediterranean countries must first identify the causes of hospital inefficiency and take necessary remedial actions to facilitate the optimal use of scarce resources.

## Author contributions

MA and HR designed the research. MA, AM-A, and PI conducted it. MA and PI extracted the data. MA, HR, VB, AM-A, and PI wrote the study. MA had primary responsibility for the final content. All authors read and approved the final manuscript.
