# Supplementary material for: Hospital efficiency in the eastern mediterranean region: A systematic review and meta-analysis
Source: Front Public Health. 2023 Feb 2;11:1085459. doi: 10.3389/fpubh.2023.1085459 (PMC9936516; doi:10.3389/fpubh.2023.1085459)
Supplement: Supplementary file 1 [file Data_Sheet_1.docx]

**Appendix**: Quality assessment tool by Mitton et al (1)


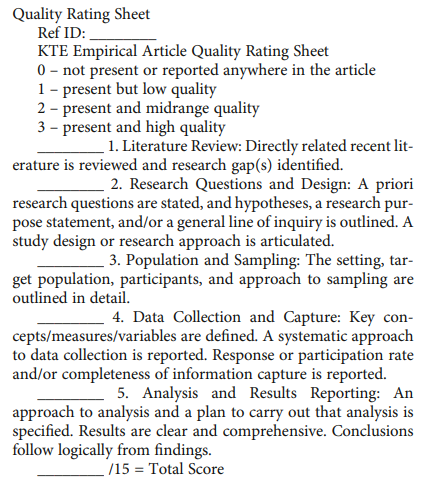


1. Mitton C, Adair CE, McKenzie E, Patten SB, Perry BW. Knowledge transfer and exchange: review and synthesis of the literature. The Milbank Quarterly. 2007;85(4):729-68.
